# Low-dose rocuronium as an adjuvant to remifentanil for laryngeal mask airway-assisted flexible fiberoptic bronchoscopy in children: protocol for a randomized placebo-controlled trial

**DOI:** 10.3389/fmed.2026.1795583

**Published:** 2026-05-08

**Authors:** Shang-xian Xu, Yu-fan Yang, Min Yang, Xiao-tian Liu, Hua-xian Liu, Hui-quan Sun, Zaixiang Tang, Ke Peng, Qian Wang

**Affiliations:** 1 Department of Anesthesiology, Children’s Hospital of Soochow University, Suzhou, Jiangsu, China; 2 Department of Anesthesiology, First Affiliated Hospital of Soochow University, Suzhou, Jiangsu, China; 3 Institute of Anesthesiology, Soochow University, Suzhou, Jiangsu, China; 4 Department of Pneumology, Children’s Hospital of Soochow University, Suzhou, Jiangsu, China; 5 Department of Biostatistics, School of Public Health, Suzhou Medical College of Soochow University, Suzhou, Jiangsu, China

**Keywords:** flexible fiberoptic bronchoscopy, laryngeal mask airway, pediatric, remifentanil, rocuronium

## Abstract

**Background:**

Flexible fiberoptic bronchoscopy is being increasingly carried out under general anesthesia in pediatric patients. However, the optimal anesthetic protocol still remains unclear. This study aims to test the hypothesis that the addition of low-dose rocuronium during laryngeal mask airway-assisted flexible fiberoptic bronchoscopy in children can enhance procedural outcomes and improve overall management.

**Methods:**

In this randomized, double-blind, placebo-controlled trial, 140 children aged 1–6 years scheduled for flexible fiberoptic bronchoscopy under general anesthesia will be randomly allocated in a 1:1 ratio to either the rocuronium group or the normal saline group (*n* = 70 in each group). All children will receive intravenous propofol at a dose of 3 mg⋅kg^–1^ and remifentanil at 3 μg⋅kg^–1^ for anesthesia induction. This will be followed by either intravenous rocuronium at 0.3 mg⋅kg^–1^ or an equivalent volume of normal saline. Sevoflurane will be titrated to achieve the target anesthesia depth, while remifentanil will be initiated at a rate of 0.05 μg⋅kg^–1^⋅min^–1^ and subsequently adjusted as required. Mechanical ventilation will be performed via laryngeal mask airway and the bronchofiberscope is inserted through an adapter connected to the laryngeal mask airway. The primary endpoint, reflecting effectiveness of anesthesia, is the frequency of coughing episodes during the operation. Secondary endpoints include: (1) cumulative remifentanil dosage; (2) failure rate of flexible fiberoptic bronchoscopy operation; (3) hemodynamic changes; (4) cough scores in the post-anesthesia care unit; (5) procedure time; (6) discharge time; (7) adverse events; (8) satisfaction scores of bronchoscopist; (9) satisfaction scores of anesthesiologist; and (10) satisfaction scores of parents 24 h after the operation.

**Discussion:**

We hypothesize that the addition of low-dose rocuronium during laryngeal mask airway-assisted flexible fiberoptic bronchoscopy under general anesthesia may optimize operation conditions, reduce coughing frequency, lower remifentanil dosage and the failure rate, stabilize hemodynamic fluctuations, minimize adverse events and enhance satisfaction, thereby providing valuable reference for clinical anesthesia practice in these procedures.

**Study Protocol Registration:**

https://www.chictr.org.cn/showprojEN.html?proj=277486, identifier [ChiCTR2500105407].

## Introduction

Flexible fiberoptic bronchoscopy (FFB) maintains its critical role in pediatric pulmonology, serving indispensable diagnostic and therapeutic functions (1, 2). The guidelines of the British Thoracic Society (BTS) suggest that sedation should be recommended for all patients undergoing bronchoscopy unless there are absolute contraindications (3–5). Reducing coughing, providing a good operating environment without compromising airway patency, and maintaining adequate oxygenation are the primary goals of anesthesiologists.

The combined use of propofol and short-acting remifentanil is the most commonly used regimen during FFB (6, 7). The antitussive and analgesic properties of remifentanil may lead to a considerable reduction in coughing, thereby optimizing the working conditions for the bronchoscopist and improving patient comfort (1, 8–10). However, coughing reactions cannot be completely suppressed even when a high dosage of remifentanil is administered, and such a large dose of remifentanil is associated with more pronounced respiratory depression. The incidence of hypoxemia approaches 30% in children undergoing FFB while receiving remifentanil (1, 6, 11–14).

The technique of FFB via the laryngeal mask airway (LMA) was first introduced in 1989 and since then it has been described as a safe and convenient tool in both adult and pediatric populations (15–18). LMA with a bronchoscopy adaptor can direct the fiberscope to the glottis and provide ventilation without interfering with the maneuver of the bronchoscopist. Thus, this technology has the potential to overcome the side effects of hypoxemia during FFB, indicating that general anesthesia may be more preferable for FFB in children (15, 19–22). However, the decision of whether to use skeletal muscle relaxants remains a significant issue that anesthesiologists need to consider (23).

Several studies, as documented in case reports, have reported positive experiences with the use of neuromuscular blocking agents in LMA-assisted FFB (19, 23). However, there is currently no established guideline for the recommendation of neuromuscular blocking agents during anesthesia for FFB. Barclay et al.’s study demonstrated that the addition of low-dose rocuronium (0.3 mg⋅kg^–1^, equivalent to the Effective Dose 95) to propofol and alfentanil significantly improved the conditions for tracheal intubation after the induction of anesthesia (24). Gelberg et al.’s study showed that the addition of 0.2 mg⋅kg^–1^ of rocuronium to propofol and remifentanil during anesthesia induction in infants results in improved intubation conditions compared to placebo, although the difference did not achieve statistical significance (25). Both of the aforementioned studies imply that low-dose neuromuscular blocking agents may be beneficial for airway operations.

In this randomized placebo-controlled study, we hypothesize that the combination of propofol, remifentanil, and 0.3 mg⋅kg^–1^ rocuronium will optimize the examination conditions, effectively decrease the frequency of coughing, minimize the necessary dose of remifentanil, stabilize the hemodynamic fluctuations, and enhance the satisfaction of bronchoscopists, anesthesiologists, and patients. This approach aims to provide a valuable reference for clinical anesthesia practices during these procedures.

## Methods

### Study design

This single-center, double-blind, randomized, placebo-controlled trial will be conducted at Children’s Hospital of Soochow University, Suzhou, China. This trial protocol follows the guidelines of Standard Protocol Items: Recommendations for Interventional Trials (SPIRIT) (Supplementary File 1).

### Ethic approval and trial registration

The study protocol received approval from the Medical Ethics Committee of Children’s Hospital of Soochow University on May 30, 2025 (Approval No. 2025018, version 3.0) and was subsequently registered at the Chinese Clinical Trial Registry (Identifier: ChiCTR2500105407) on July 03, 2025. https://www.chictr.org.cn/showprojEN.html?proj=277486.

### Patients

**FIGURE 1:**
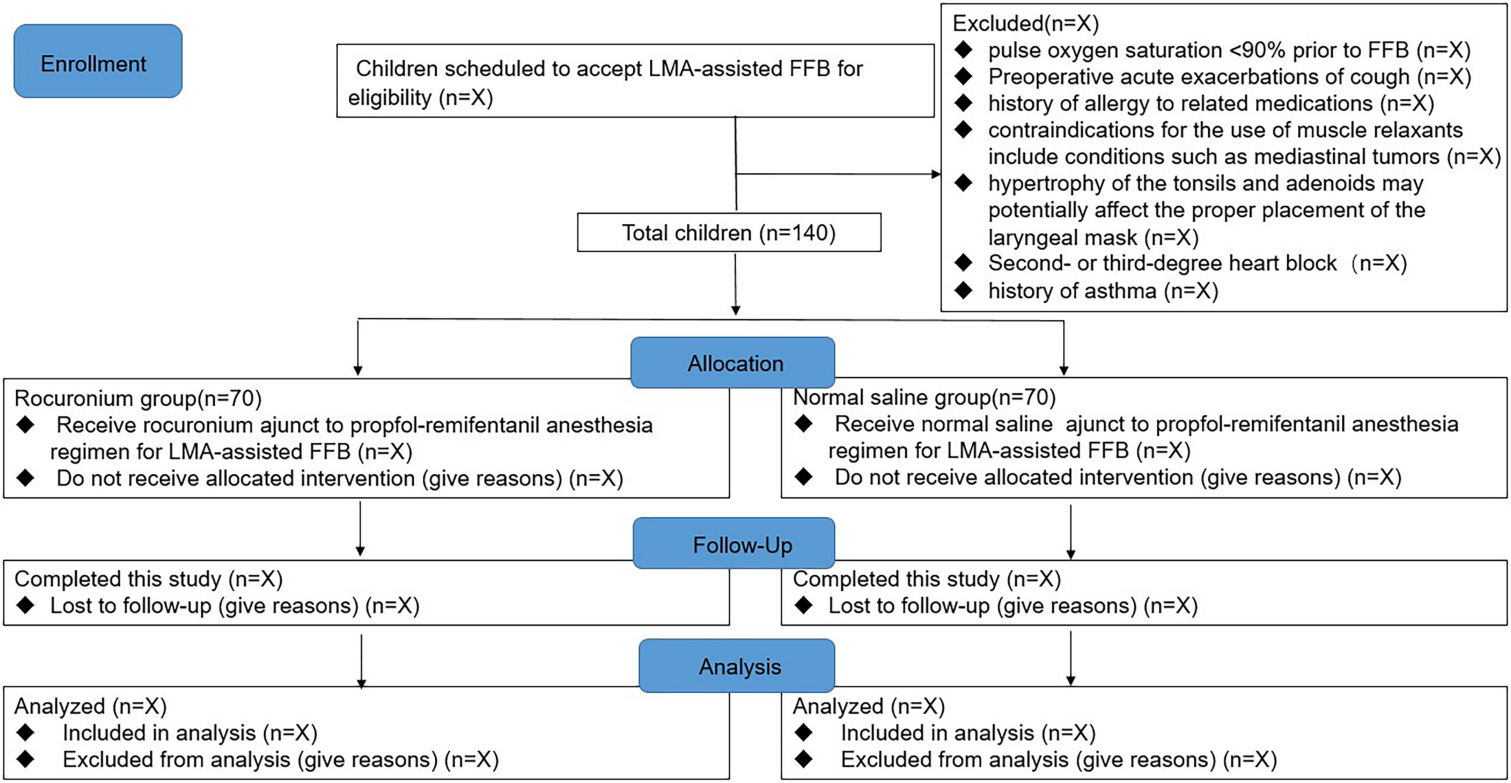
Flow chart of patients in this study. FFB, flexible fibreoptic bronchoscopy; LMA, laryngeal mask airway.

An experienced anesthesia assistant will conduct a comprehensive screening of all the children to determine their eligibility and communicate with the parents or guardians of the children to encourage their active participation in the research. The principal and associate investigators will personally present the Participant Information and Consent Form in the guardian version, clearly delineating the study’s purpose, procedures, benefits, risks, and confidentiality policies. Additionally, they will provide the Participant Information and Consent Form in the children’s version, which is written in plain and age-appropriate language. They will also explicitly clarify the participants’ right to withdraw from the study at any time without incurring any adverse consequences. To be included in this study, children must meet the eligibility criteria, and their parents or guardians should provide written informed consent. A total of 140 eligible children scheduled for FFB under general anesthesia will be randomly assigned to either the rocuronium group or the placebo group, with 70 children in each group. The flow chart of this study is presented in Figure 1.

### Eligibility criteria

Inclusion criteria: (1) Aged between 1 and 6 years; (2) American Society of Anesthesiologists (ASA) physical status of II or III; (3) body mass index (BMI) between 14 and 18 kg/m^2^; (4) scheduled for FFB under general anesthesia due to conditions such as pneumonia, broncho-alveolar lavage (BAL), airway mucosal biopsy, or foreign body removal.

Exclusion criteria: (1) pulse oxygen saturation (SpO_2_) < 90% before FFB; (2) preoperative acute exacerbations of cough which is specifically defined as a clear increase in cough frequency to approximately twice that of the patient’s typical baseline pattern during the current illness, as reported by the parent/guardian, and this increase is not a feature of the child’s illness in the preceding days. This assessment will be guided by using a simple categorical scale: “Is your child coughing noticeably more often today than they were 2 days ago? Would you estimate it as “a little more,” “about twice as much,” or “much more than twice as much?”” A report consistent with “about twice as much” or “much more than twice as much” will meet this criterion; (3) history of allergy to relevant medications; (4) contraindications for the use of muscle relaxants such as mediastinal tumors; (5) hypertrophy of the tonsils and adenoids that may potentially affect the proper placement of the laryngeal mask; (6) second- or third-degree heart block; (7) history of asthma.

### Randomization and blinding

An independent biostatistician, not involved in data management or analysis, generates the randomization list using a validated online tool^1^. The randomization is carried out using a 1:1 allocation ratio and permuted blocks of sizes 2 and 4. The allocation sequence will be executed using a password-protected, web-based randomization module. The encrypted database can be accessed only by an unblinded trial nurse. Based on the randomization list, the nurse will prepare and distribute the study medications (rocuronium or normal saline placebo) in identically labeled syringes marked only with study numbers. It is visually impossible to distinguish the contents of these syringes because both rocuronium and normal saline are clear and colorless solutions. Patients, endoscopists, outcome observers, researchers responsible for recording the data, and data analysts will all be kept blinded to the group assignment until the final analysis is finished. Due to safety considerations and the necessity of monitoring the train-of-four (TOF) ratio, the primary anesthetist, responsible for administering anesthesia to the patients as well as monitoring the TOF ratio at the ulnar nerve, is unblinded to the group assignments. However, the primary anesthetist neither knows the hypothesis of this study nor participates in outcome assessment. The patient recruitment, study interventions, and outcome measurement schedule are presented in Table 1.

**TABLE 1:** Schedule of patient enrollment, study intervention, and endpoint measurement.

Timepoint	Study period				
	Enrollment	Allocation	Post-allocation		
	Preanesthetic visit	Before operation	During operation	PACU	24 h after the operation
Enrollment	×	×			
Inclusion criteria	×				
Exclusion criteria	×				
Written informed consent	×				
Baseline characteristics	×				
Preoperative cough status	×				
Randomization					
Allocation		×			
Intervention					
Group rocuronium			×		
Group normal saline			×		
Endpoint measures					
Frequency of coughing episodes			×	×	×
Cumulative remifentanil dosage			×		
Failure rate			×		
Hemodynamic variables			×		
Cough scores in the PACU			×		
Procedure time				×	
Discharge time			×		
Adverse events				×	
Satisfaction scores of bronchoscopist			×	×	
Satisfaction scores of anesthesiologist					
Satisfaction scores of parents					

According to SPIRIT 2013 statement of defining standard protocol items for clinical trials. PACU, post-anesthesia care unit.

### Study interventions

#### Anesthesia preparation stage

All children are screened for eligibility by an experienced anesthesia assistant who will not be involved in block sizes and future allocation. Baseline data including heart rate (HR), systolic blood pressure (SBP), diastolic blood pressure (DBP), pulse oxygen saturation (SpO_2_) are recorded. Children with a diagnosis of pneumonia or airway foreign body but with a stable, improving, or predictable pattern of cough will be eligible for inclusion. Their baseline cough frequency (“occasional” or “frequent but stable”) will be recorded as a characteristic for group comparison. Children are restricted from consuming solid foods for 6 h and clear fluids for 2 h prior to undergoing FFB (26). A patent intravenous cannula is inserted, and premedication with intravenous midazolam at a dose of 0.05 mg⋅kg^–1^ is given in the ward. Topical anesthesia is achieved by administering a 2-milliliter nebulized 2% lidocaine solution through inhalation before the procedure in the ward.

#### Anesthesia induction stage

All FFB procedures are carried out in the outpatient bronchoscopy room with children positioned in a supine position. Throughout the procedures, HR, end-tidal partial pressure of carbon dioxide (PetCO_2_), SpO_2_, body temperature and the bispectral index (BIS) are continuously monitored and noninvasive blood pressure (NIBP) is measured at 3-min intervals. Anesthesia depth is monitored using a combination of standard clinical signs and BIS. The BIS is a dimensionless number ranging from 0 to 100, which is calculated based on frontal electroencephalographic signals. The primary goal is maintaining clinical signs of adequate anesthesia, defined by the absence of purposeful movement or gross sympathetic response (tachycardia > 20% above baseline or hypertension > 20% above baseline). The BIS value of 40–60 serves as a secondary guide, which is intended as an adjunctive, quantitative tool to minimize the risk of awareness and help maintain a relatively stable hypnotic state. A nasopharyngeal temperature probe is promptly placed after the induction of anesthesia, and temperature readings are documented at 3-min intervals throughout the entire case. Normothermia is precisely defined as a core temperature ranging from 36.0 °C to 37.5 °C. To effectively maintain normothermia, a forced-air warming blanket that is set at a temperature between 32 °C and 43 °C is available if needed. In the event that body temperature falls below 36.0 °C, the forced-air warmer temperature is increased. No patient is discharged from the operating room with a body temperature < 35.5 °C. TOF ratio is monitored immediately prior to the insertion of LMA and upon the completion of the procedure. All participants receive anesthesia that is administered by the same primary anesthesiologist. Following the intravenous administration of propofol at a dosage of 3 mg⋅kg^–1^, TOF stimulation is delivered at 15-s intervals. After 2 min of stable readings, the baseline twitch height is set to 100% for calibration. Then, remifentanil is administered at a dosage of 3 μg⋅kg^–1^. As spontaneous breathing gradually subsides, manual mask ventilation with 100% oxygen is provided as a supportive measure. Subsequently, an intravenous dosage of rocuronium at 0.3 mg⋅kg^–1^ is administered to the rocuronium group, while an equivalent volume of normal saline is given to the normal saline group. Immediately after the administration of all the induction drugs, remifentanil is infused at an initial rate of 0.05 μg⋅kg^–1^⋅min^–1^ (7). After 60 s of the administration of rocuronium or normal saline, the TOF ratio is recorded, and an appropriately sized LMA is inserted. The LMA is then connected to an anesthesia machine to manage ventilation in synchronized intermittent mandatory ventilation mode with a tidal volume of 6 ml/kg and adjust the breathing rate to maintain PetCO_2_ at 30–40 mmHg. Oxygen is delivered at a concentration of 50% and a flow rate of 2 L/min. Meanwhile, sevoflurane is titrated with an initial concentration of 1.5% through the LMA to maintain the appropriate anesthesia depth. If the clinical signs are adequate but the BIS values persistently exceed 60, the concentration of sevoflurane is increased in 0.5% increments. Conversely, if the BIS values are within or below the target range but the clinical signs suggest light anesthesia (such as movement, tachycardia or hypertension), the clinical signs take precedence, and the concentration of sevoflurane is increased in 0.5% increments. No reduction in the sevoflurane concentration is made solely based on a low BIS value if the clinical assessment indicates an adequate depth of anesthesia.

#### During the procedure

An Olympus BF-XP290 flexible fiberoptic bronchoscope with an outer diameter of 2.8 mm, or a BF-P290 with an outer diameter of 4.0 mm is inserted through the bronchoscope connector. The suction port is sealed with a Polyvinyl Chloride cover to create an airtight seal around the bronchoscope. When the vocal cords are visualized, 2% lidocaine, usually administered in a volume of 1 mL, is applied to the vocal cords via the bronchoscope to prevent laryngospasm. Then, the vocal cords are carefully traversed, and 1 ml of 2% lidocaine is instilled into the tracheobronchial tree using the “spray as you go” technique (27). Further diagnosis and treatment of the airway using a flexible bronchoscope is performed based on the specific clinical context. The infusion rate of remifentanil is adjusted every minute, increasing or decreasing by 0.01 μg⋅kg^–1^⋅min^–1^ based on any signs of coughing. If a cough reaction occurs, the infusion rate is increased; if no cough reaction is observed, the infusion rate is decreased, as determined by the primary anesthesiologist. Supplemental boluses of 1–2 μg⋅kg^–1^ remifentanil will be administered as a remedy and repeated as needed in the event of persistent coughing. If blood pressure or other hemodynamic parameters preclude the administration of adequate doses of remifentanil, a supplementary dose of rocuronium (0.2 mg⋅kg^–1^) is administered. Cases in which a supplementary dose of rocuronium is administered are defined as those “with the highest incidence of coughing” and are categorized as “failure cases.”

#### Anesthesia recovery stage

Upon completion of the procedure, discontinue all medications. Remove the LMA once spontaneous breathing has fully recovered and the TOF ratio reaches 0.9. Although the protocol employs a low dose of rocuronium, and spontaneous recovery to a TOF ratio ≥ 0.9 is anticipated within 20–30 min in most children, sugammadex is readily available and will be administered at the end of the procedure if the TOF ratio remains <0.9 or if there are clinical signs of residual neuromuscular blockade such as inadequate tidal volume. The decision to reverse is made by the primary anesthesiologist. The dose of sugammadex will be 2 mg⋅kg^–1^ if the TOF ratio is <0.9, a dose sufficient for rapid reversal of low-dose rocuronium-induced moderate neuromuscular blockade while avoiding unnecessary higher-dose exposure. If spontaneous recovery has already achieved a TOF ratio ≥ 0.9, no reversal agent will be given. The usage, dosage and the corresponding TOF ratio at the time of reversal will be recorded. Following the removal of the LMA, children will be transferred to the post-anesthesia care unit (PACU). Hemodynamic data (HR, SBP, DBP), SpO_2_ and coughing scores are monitored every 5 min until children are discharged. Coughing scores is defined according to the frequency of coughing episodes: Grade 0 (no coughing), Grade 1 (minimal: once or twice), Grade 2 (moderate: 3–4 times), and Grade 3 (severe: 5 or more times). Children are ready for discharge when they are fully awake and have reached a modified Aldrete score of ≥9.

### Primary endpoint

The primary endpoint is the frequency of coughing episodes during FFB, calculated as the total number of coughing episodes divided by the procedure time. One coughing episode is defined as a period of continuous coughing that interferes with either adequate ventilation or procedural progress (1). Interference with ventilation is primarily identified by the main observer through real-time visual inspection of the capnography waveform and ventilator parameters. It is characterized by flattening or significant distortion of the capnography waveform for ≥3 s coincident with the cough or visible, sustained chest wall movement against the set ventilator cycle coincident with the cough. A concomitant decrease in SpO2 of ≥5% is a supportive but not mandatory criterion. Procedural interference refers to a situation where the bronchoscopist will verbally confirm any instance where coughing necessitates a deliberate pause in the advancement or manipulation of the bronchoscope to maintain visual clarity. A cough-free interval of at least 3 s between two consecutive coughs is considered as the criterion for distinguishing two separate coughing episodes.

Addressing the inherent challenge of precise chronometry during a dynamic procedure, the primary endpoint assessment will be conducted in real-time by a dedicated observer who is not engaged in other procedural tasks. This observer will receive training on the assessment protocol prior to the start of the trial to standardize recognition. The dedicated observer will also be trained to identify a distinct, perceptible pause in the coughing paroxysm, consistent with the approximate duration of 3 s, before recording a new episode. This method prioritizes clinical relevance and feasibility during an active procedure.

### Secondary endpoints

The secondary endpoints are: (1) cumulative remifentanil dosage; (2) success rate of FFB operation; (3) hemodynamic variables; (4) cough scores in the PACU every 5 min; (5) procedure time defined as the duration from bronchoscope insertion to its removal; (6) discharge time which is defined as the interval from the removal of bronchoscopy until a modified Aldrete score of ≥9 is achieved; (7) adverse events that occur during the procedure and in the PACU, including desaturation (SpO_2_ < 90%) which is stratified into three categories: mild desaturation: 85% ≤ SpO2 < 90%, moderate desaturation: 80% ≤ SpO2 < 85%, severe desaturation: SpO2 < 80%; laryngospasma; sore throat; bradycardia or hypotension defined as a decrease of at least 20% below the age-adjusted lower limit of heart rate or arterial blood pressure; tachycardia or hypertension defined as an increase of at least 20% above the age-adjusted upper limit of heart rate or arterial blood pressure; (8) satisfaction scores of bronchoscopists during the operation; (9) satisfaction scores of anesthesiologists; (10) satisfaction scores of parents 24 h after the operation. Given the age range of our study population (1–6 years), this score is reported as a proxy measure by the child’s parent or guardian, who is present throughout the preoperative, recovery, and immediate post-discharge periods. Satisfaction scores are measured subjectively by a simple numeric score between 1 (highly dissatisfied) and 10 (highly satisfied).

### Data collection and management

Demographic data include sex, age, weight, height, and BMI. Baseline characteristics encompass ASA physical status, as well as baseline values for HR, NIBP, SpO_2_, and baseline cough frequency (“occasional” or “frequent but stable”). Periprocedural data include the following: the total number of coughing episodes during the FFB intervention period; procedure time; cumulative remifentanil dosage; success rate of the FFB operation; HR, NIBP, SpO_2_, PetCO_2_, body temperature, BIS value, severity grading of pneumonia; the usage and dosage of sugammadex, the corresponding TOF ratio at the time of reversal; cough scores recorded in the PACU; discharge time; and adverse events that occurs during the procedure and in the PACU, including desaturation (SpO_2_ < 90%), laryngospasm, sore throat, bradycardia or hypotension, tachycardia or hypertension. Additionally, the data will capture anesthesiologist satisfaction, bronchoscopist satisfaction and parent satisfaction. All raw data will be meticulously documented in the Case Report Forms (CRFs) by an independent investigator. Upon completion of the trial for all patients, the principal investigator and the data administrator will jointly verify the integrity and accuracy of the data. Subsequently, the data administrator will import the verified data into a designated electronic database and forward it to a statistician for final statistical analysis.

#### Sample size

In a previous study by Tschiedel et al. comparing the use of propofol alone versus propofol combined with remifentanil during FFB, the addition of remifentanil resulted in a reduction of coughing episodes by approximately 60%, from 1.98 to 0.73 episodes per minute (1). Before implementing this trial, we prospectively studied 20 children undergoing FFB from April 2025 to May 2025. Our findings revealed that the frequency of cough episodes in children using the propofol-remifentanil regimen ranged from 0.6 to 0.89 episodes per minute, with a standard deviation (SD) of 0.3 episodes per minute, which is consistent with previously published data. Another 50% reduction in coughing episodes, decreasing the frequency to 0.3 episodes per minute with rocuronium, represents not only a statistically significant difference but also a clinically relevant improvement. We will require 64 subjects per group to achieve a power of 0.80 with a type I error rate of 0.05. Accounting for an anticipated dropout rate of 10%, the adjusted estimated sample size will be 70 children per group, resulting in a total of 140 children being randomized.

#### Statistical analysis

All data will be checked for normal distribution using the Kolmogorov test. For normally distributed data, continuous variables will be presented as mean ± SD and analyzed using the independent Student’s *t*-test. Nonnormally distributed data will be presented as the median and interquartile range (IQR) and compared using the Mann-Whitney U test where appropriate. Categorical data are presented as numbers (proportions, %) and will be analyzed using Fisher’s exact test or the chi-square test. For the study endpoints, 95% confidence intervals will be reported as appropriate to provide a more comprehensive understanding of the results.

The primary endpoint is the frequency of coughing episodes during the FFB operation period. If the primary endpoint adheres to a Poisson distribution or a negative binomial distribution, negative binomial regression is further utilized, adjusting for the influence of covariates [the severity classification of pneumonia, BMI, age, sex and operation time, baseline cough frequency (“occasional” or “frequent but stable”)]. If the primary endpoint conforms to a normal distribution, a multiple linear regression model is further adopted for the same covariate adjustment. Subgroup analyses for the primary endpoint will be performed according to age (<3 years old vs. ≥3 years old), sex (female vs. male), BMI (<15 vs. ≥15), operation type (BAL vs. BAL + airway mucosal biopsy vs. foreign body removal) and operation time (<20 min vs. ≥20 min). Recognizing the possibility of intra-observer variability in applying the cough threshold, we conducted a pre-specified sensitivity analysis for the primary endpoint. The frequency of coughing episodes will be re-calculated using two alternative, stricter definitions: “Worst-case” consolidation and “Best-case” granularity. The conclusions of the primary analysis are considered robust only if the between-group difference (rocuronium vs. normal saline) remained statistically significant (*p* < 0.05) in the same direction under both alternative sensitivity analysis scenarios.

The secondary endpoints will be analyzed using the multivariate logistic regression model or the generalized linear model, adjusting for the same covariates as those of the primary endpoint. Repeated measurement data will be analyzed using a mixed linear model to compare haemodynamic variables and cough scores in the PACU between groups. To address the issue of multiple comparisons for adverse events observed in secondary endpoints, the Benjamini-Hochberg procedure will be employed to control the false discovery rate.

The analysis will be performed on the modified intention-to-treat population. To ensure robustness, a per-protocol analysis will additionally be conducted; however, the intention-to-treat approach will remain the primary basis for analysis. Missing data will be processed by multiple imputation and maximum likelihood method. All endpoints are performed at the two-sided 5% significance level. Statistical analyses will be performed using SPSS statistics (version 19.0; IBM SPSS, Chicago, IL, USA).

## Discussion

This randomized controlled trial will recruit a total of 140 children to evaluate the effects of low-dose rocuronium compared with normal saline placebo on the incidence of coughing during the FFB procedure. In addition, we will compare the cumulative remifentanil dose, the success rate of the FFB operation, hemodynamic variables, cough scores in the recovery room, procedure time, adverse events during the procedure and in the PACU (including desaturation, laryngospasm, sore throat, bradycardia or tachycardia, hypertension or hypotension), discharge time and satisfaction scores of the anesthesiologist, bronchoscopists and patients. We also monitor the neuromuscular function using TOF ratio after administration of 0.3 mg⋅kg^–1^ rocuronium, as well as at the end of the procedure.

Stimulation of the larynx and trachea, particularly in patients under light anesthesia, may elicit intense coughing and potentially lead to laryngospasm (7). Remifentanil, a short-acting opioid, represents an especially promising choice for managing coughing during flexible bronchoscopy procedures. Li et al. reported that remifentanil can be safely and effectively administered to children undergoing flexible bronchoscopy (9). Compared with traditional sedation using only propofol, the combination of remifentanil and propofol for FFB results in a significant reduction in coughing episodes, thereby improving procedural tolerance and patient comfort. Despite these benefits, the use of remifentanil may lead to cardiorespiratory complications (14). Studies have demonstrated that the incidence of such complications varies between 20% and 60%, depending on the type of procedure and the chosen medication regimen (6, 28).

The utilization of LMA for airway management during FFB was first introduced in 1989. Since then, it has been widely recognized as a safe and effective device for airway control in both adult and pediatric patients (16). Alon et al.’s study in adults demonstrated that the use of the LMA provides superior airway support, maintains stable oxygen saturation, and offers a more convenient access point during FFB (16). Suzen et al. demonstrated that performing flexible bronchoscopy via an LMA is a safe, straightforward and efficient technique for the management of tracheobronchial foreign bodies in pediatric patients (22). Eliezer and colleagues also reported the safety of using the LMA during FFB. None of their patients experienced any complications or disturbances in gas exchange, and oxygen saturation levels were consistently maintained within the range of 95%–100% (20).

Neuromuscular blocking agents are widely used as an anesthesia adjunct to facilitate tracheal intubation and mechanical ventilation, relax abdominal muscles, and ensure immobility during surgery (29, 30). Tips and tricks for anesthesia during pediatric rigid bronchoscopy indicate that neuromuscular blocking agents can be administered when blood pressure or other hemodynamic parameters preclude the administration of sufficient doses of anesthetic agents (31). In Dich et al.’s study, which involved 20 adult participants, total intravenous anesthesia with suxamethonium was demonstrated to be a safe technique for LMA-assisted FFB (19). A retrospective observational study conducted by Li et al. demonstrated that muscle relaxants and low-frequency conventional ventilation can be safely utilized in both flexible and rigid bronchoscopy procedures for patients with chronic airway obstruction. They found that no patients experienced ventilation failure, bronchospasm, or intraoperative coughing (23). The role of neuromuscular blocking agents in FFB remains incompletely understood. To our knowledge, this will be the first randomized controlled trial to evaluate whether the adjunctive use of low-dose rocuronium in combination with remifentanil during LMA-assisted FFB in children leads to reduced coughing, a lower remifentanil dosage, more stable hemodynamics, no impact on discharge time, and increased satisfaction among anesthesiologists, bronchoscopists and parents. The selection of rocuronium over cisatracurium represents a deliberate choice based on the specific requirements of this trial. Rocuronium at 0.3 mg⋅kg^–1^ provides a rapid-onset, moderate-depth neuromuscular blockade whose duration approximates the typical procedure time. Given the availability of sugammadex, the duration of action becomes both flexible and controllable. Any residual blockade at the end of the procedure can be fully reversed within 2–3 min. However, we acknowledge that cisatracurium offers theoretical advantages, including organ-independent metabolism and a recovery profile less influenced by temperature or hepatic and renal function. In the context of this study conducted in healthy children undergoing brief procedures with active temperature management, these advantages are considered less critical than the practical benefits of rocuronium. In addition to reducing coughing and providing improved operating conditions, the use of low-dose rocuronium may allow for a reduction in the doses of remifentanil required to achieve adequate procedural conditions. Such a reduction could translate into faster emergence from anesthesia, fewer respiratory complications in the PACU, and an overall enhanced recovery profile. For our pediatric patients, intravenous propofol at a dose of 3 mg⋅kg^–1^ in combination with remifentanil at 3 μg⋅kg^–1^ will be utilized for induction. This will be followed by the administration of either 0.3 mg⋅kg^–1^ of rocuronium or an equivalent volume of normal saline. Sevoflurane will be titrated to achieve the appropriate depth of anesthesia, while remifentanil will be continuously infused. A LMA with an interface is inserted to guide the fiberscope passage while allowing uninterrupted ventilation and maintaining oxygenation.

This study has several limitations. First, despite variations in operation times, with some even exceeding the metabolic time of rocuronium, a uniform dose of 0.3 mg⋅kg^–1^ will be administered to all children. This decision was made to minimize the risk of delayed spontaneous breathing recovery due to incomplete metabolism of muscle relaxants. Second, children younger than 1 year will be excluded, as the cross-sectional area of the airflow channel in the LMA designed for this age group may be insufficient to accommodate the flexible bronchoscope. Third, the initial real-time assessment on coughing episode brings about the possibility of observer bias. We alleviated this issue by using predefined, measurable criteria and having the assessment conducted by a single-blinded observer. Nevertheless, the potential for variability in clinical judgment still remains a limitation of the assessment method. The operational definition of the cough-free interval, while clear in principle, presents a practical measurement challenge. To mitigate the impact of potential misclassification, we employed pre-planned sensitivity analyses using extreme re-definitions of an episode. The consistency of our primary finding across these alternative analyses supports the conclusion that any measurement noise introduced by the real-time application of the 3-s rule is unlikely to account for the observed treatment effect. Fourth, our protocol utilized BIS monitoring as an adjunct to clinical assessment. While this provides an additional objective parameter, the interpretation of BIS values in children requires caution. The algorithm’s validation is less established in children than in adults, and values can be influenced by physiological and pharmacological factors not necessarily indicative of cortical suppression of airway reflexes. Our hierarchical management protocol, which prioritized clinical signs over BIS values, is designed to mitigate this limitation. However, the inherent variability and potential inaccuracy of BIS in this pediatric population mean it could not serve as a perfect surrogate for reflex suppression, and this constitutes a limitation of the monitoring methodology. Fifth, patient satisfaction is reported by parents or guardians rather than by the children themselves, it represents a proxy assessment of the caregiver’s perception of the child’s experience and their own experience of the process. While this is a common and practical approach in pediatric research involving young children, it may not directly correlate with the child’s self-assessed experience, especially for non-verbal or pre-verbal children. Therefore, while this measure provides valuable insight into a key stakeholder’s perspective, it should not be equated with patient-reported outcomes in older, cognitively capable populations. This study enrolls children with active pneumonia or airway foreign body, nearly all of whom may present with a cough. To isolate the effect of the intervention on procedure-induced coughing, we exclude children who have an acute, subjective exacerbation of cough (a ≥2-fold increase) prior to the procedure. This operational definition, although relying on parental report, offers a practical threshold for identifying children with unstable airway symptoms. Nevertheless, the baseline level of airway inflammation and irritability caused by pneumonia or foreign body itself remains a potential confounding variable. We record the baseline cough frequency to ensure balance between groups through randomization and set it as a covariate. We also acknowledge that the background “cough propensity” stemming from the underlying disease is an inherent limitation when studying this clinically relevant endpoint. Even with its predefined constraints, this study will provide the first randomized evidence on the efficacy and safety of this specific adjuvant technique. The findings from this trial should pave the way for more nuanced investigations. Future studies could explore weight- or time-adjusted dosing regimens of rocuronium to optimize the balance between efficacy and recovery. Furthermore, our work highlights the need for the development of specialized LMAs with larger airway channels suitable for infants, which would allow subsequent research to include this vulnerable age group and create a comprehensive clinical guideline across all pediatric ages.

## Footnotes

1
https://www.sealedenvelope.com/simple-randomiser/v1/lists
